# A bibliometric analysis of research on terrestrial isopods

**DOI:** 10.3897/zookeys.1101.81016

**Published:** 2022-05-18

**Authors:** Miloš Vittori, Miha Dominko

**Affiliations:** 1 Department of Biology, Biotechnical Faculty, University of Ljubljana, Večna pot 111, SI-1000 Ljubljana, Slovenia University of Ljubljana Ljubljana Slovenia; 2 Institute for Economic Research, Kardeljeva ploščad 17, 1109 Ljubljana, Slovenia Institute for Economic Research Ljubljana Slovenia

**Keywords:** Citation analysis, crustacean, invertebrate zoology, Oniscidea, scientometrics

## Abstract

Terrestrial isopods (Oniscidea) are crustaceans that thrive in terrestrial environments. This study provides an overview of the major topics in terrestrial isopod research during the last 70 years in order to provide an example of publication practices in invertebrate zoology and to examine how basic research in this area is transferred to its applications. Co-citation analysis and bibliographic coupling based on citation data from the Web of Science Core Collection was used. Findings show that while research on terrestrial isopods expanded in applicative research prioritised by research policies, basic research continues to flourish. The most productive countries in the field include the major developed economies and several smaller nations. In the smaller countries, as well as in France and Italy, the bulk of woodlouse research is performed at a few institutions with traditions in this field. Some of the most influential works have been published in periodicals or monographs that are not indexed in Web of Science or Scopus and lack impact factors. Conference proceedings represent some of the most influential publications in the field. Our findings indicate that smaller and developing economies make significant contributions in invertebrate zoology if their research organisations can achieve continuity of research on a topic. Another conclusion is that journal metrics may be a misleading descriptor of the impact of studies and researchers in this field. Ultimately, these results identify several examples of how basic research in invertebrate zoology leads to applications with considerable socio-economic impact.

## Introduction

Invertebrate zoology, as a scientific field, studies the greater part of all living animals, as invertebrates represent the majority of animal biodiversity. Basic research in this field forms the foundation for applicative studies that may extend to other scientific fields and is essential to conservation efforts. Nevertheless, basic research of invertebrates other than model organisms receives relatively little funding and little attention in educational curricula (Leather 2009; Martin 2011). This puts invertebrate zoology in a paradoxical position: although we might expect it to be an extremely important field in the broader scope of the life sciences, it is perceived by the public as almost completely irrelevant. For this reason, it is important to understand how knowledge is shared in the community of researchers in this field and how it is ultimately transferred to its applications.

**Figure 1.**
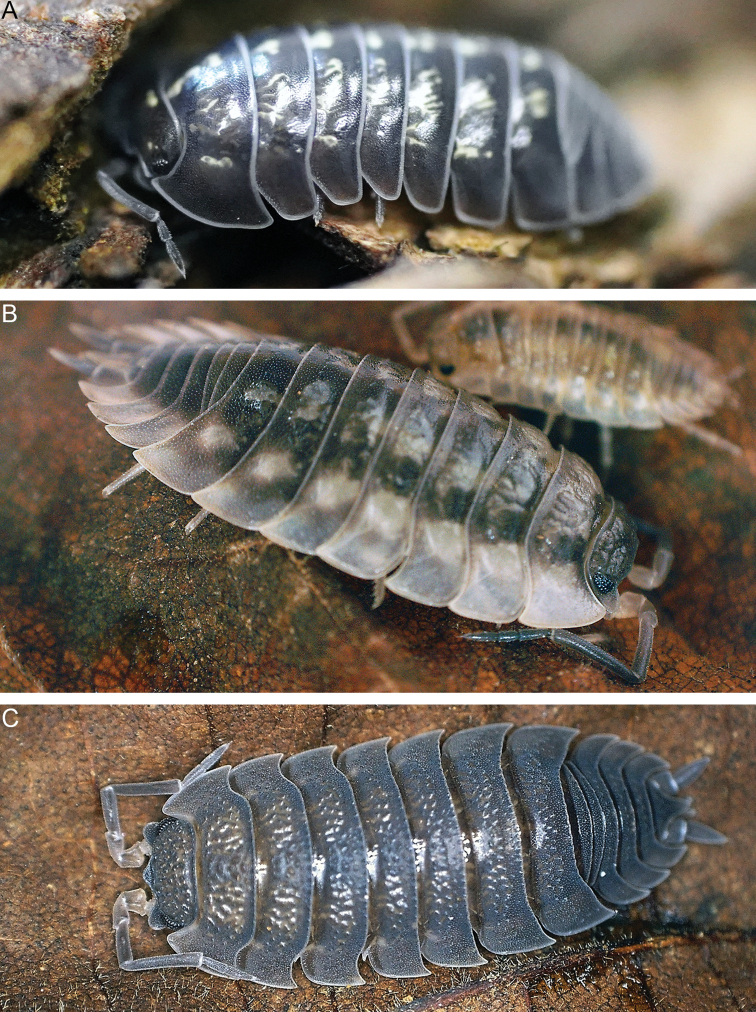
The three most extensively studied woodlouse species (Schmalfuss 2003) **A***Armadillidiumvulgare***B***Oniscusasellus***C***Porcellioscaber*. Photographs by Ana Sterle (**A**) and Miloš Vittori (**B, C**).

Terrestrial isopods or woodlice (Oniscidea) are a group of crustaceans that has successfully adapted to the terrestrial environment (Fig. 1). The most recent published account of their diversity lists more than 3700 recognised species, a number that has certainly grown since, making woodlice the most diverse group of isopod crustaceans (Sfenthourakis and Taiti 2015). As litter decomposers, these animals have a profound ecological impact (David and Handa 2010). Woodlice display various degrees of terrestrialisation, making them particularly interesting to the study of evolutionary transition to land (Hornung 2011). In addition to the studies of their diversity and ecology, research interest in terrestrial isopods is very broad, ranging from biogeography to ethology and biochemistry (Schmalfuss 2018).

A comprehensive list of literature on terrestrial isopods published prior to 2000 was assembled by Schmalfuss and Wolf-Schwenninger (2002). The list covers scientific publications dealing specifically with the biology of terrestrial isopods. Schmalfuss (2018) also provided a historic overview of prominent researchers working on terrestrial isopods, from Aristotle to currently active research groups. His survey focused predominantly on research in the field of isopod systematics and the lives and work of some of the giants of woodlouse systematics in the 20^th^ century. It also presented the work of prominent researchers working on other aspects of terrestrial isopod biology. An account of major topics in terrestrial isopod biology was also given in the most recent review of the state of knowledge on these crustaceans, published by Hornung (2011).

In the present contribution, we use bibliometric methods to obtain an overview of the main topics and trends in research dealing with terrestrial isopods, including fields of research that use woodlice as experimental organisms and do not necessarily focus on the biology of this taxonomic group as such. Our aim is to use terrestrial isopod research as a case study of how invertebrate zoology functions in the modern scientific environment and to outline how basic research on invertebrates is linked to its applications. We quantitatively describe the development of this field during the second half of the 20^th^ century and the first two decades of the new millennium and identify the studies that influenced the development of various research directions in the field, including an account of what types of publications they were published in.

## Materials and methods

Bibliometrics are becoming an integral part of research evaluation due to the greater availability of article and citation data, as well as the development of new analysis software (Ellegaard and Wallin 2015). Compared to traditional review techniques, they offer several advantages. Firstly, they are a quantitative way of measuring research impact, meaning that they are objective. Secondly, they are transparent and the results can be replicated using the same method. Thirdly, they are scalable, which means that they can be applied on an individual, institutional, national, or international level. Finally, they allow for the analysis of publication performance, as well as the structure and dynamics of the research field under study.

Our bibliometric analysis relies on citations, which provide an objective measure of a paper’s impact in a field of knowledge (Garfield 1979). We use two methods, namely co-citation analysis and bibliographic coupling (Kessler 1963; Small 1978). Each uses citation relationships between publications in its own way, thus complementing each other. Co-citation analysis clusters publications that are often cited together. This property allowed us to identify important publications not included in our database due to being published in non-indexed journals or books as well as works published earlier than the publications in the database. In contrast to co-citation, bibliographic coupling clusters publications with overlapping bibliographies and is thus an adequate technique for the detection of the current state of research, as well as the identification of future trends.

To obtain a relevant dataset of publications on terrestrial isopods, we used the Web of Science Core Collection, which contains publication and citation data. We obtained our dataset by searching for the scientific and trivial names of the taxon Oniscidea, as well as several of the most studied genera of terrestrial isopods that we were able to identify in the World catalogue of terrestrial isopods (Schmalfuss 2003) and the literature overview by Schmalfuss and Wolf-Schwenninger (2002). Our search was limited to the period between 1950 and 2020. While it is possible to search WoS for works published as far back as 1900, few works published before 1950 are indexed in WoS and including this period would not be representative. Earlier relevant publications were therefore identified using co-citation analysis. Upon inspection of the obtained database, we refined the search by excluding keywords most frequently shared by publications that were not relevant to the study.

To obtain our dataset we applied the following search using the appropriate Boolean operators (AND, OR, and NOT):

- TS=”terrestrial isopod*” OR TS=”oniscoid*” OR TS=”woodlice” OR TS=”oniscid*” OR TS=”pill bug” OR TS=”sow bug” OR TS=”sea slater” OR TS=”roly-poly” OR TS=”potato bug” OR TS=”armadillidium” OR TS=”porcellio*” OR TS=”philoscia” OR TS=”oniscus” OR TS=”ligia” OR TS=”hemilepistus” OR TS=”platyarthrus” OR TS=”woodlouse”
- TI=”crab” OR TI=”crayfish” OR TI=”decapod” OR TI=”daphnia” OR TI=”aquatic” OR TI=”amphipod*” OR TI=”lobster” OR TI=”prawn” OR TI=”shrimp” OR TI=”gastropod*” OR TI=”snail” OR TI=”marine isopod” NOT (TI=”terrestrial” OR TI=”littoral”)
- TS=”mitochondrial” OR TS=”androgenic gland” NOT (TS=”terrestrial isopod*” OR TS=”oniscoid*” OR TS=”woodlice” OR TS=”oniscid*” OR TS=”pill bug” OR TS=”sow bug” OR TS=”sea slater” OR TS=”roly-poly” OR TS=”potato bug” OR TS=”armadillidium” OR TS=”porcellio*” OR TS=”philoscia” OR TS=”oniscus” OR TS=”ligia” OR TS=”hemilepistus” OR TS=”platyarthrus” OR TS=”woodlouse”)
- TS=”random walk” OR TS=”navigation” OR TS=”path integration”
- #1 NOT (#2 OR #3 OR #4)

To visualise bibliometric networks, we used VOSviewer (van Eck and Waltman 2010) and CitNetExplorer (van Eck and Waltman 2014a). VOSviewer can visualise networks of keywords, individual publications, authors, journals, or even countries based on citation, bibliographic coupling, co-citation, and co-authorship relations. To structure a bibliometric map, VOSviewer first uses a co-occurrence matrix to obtain a similarity matrix. Moreover, it constructs a map by locating items close to each other by minimising the weighted sum of the squared Euclidean distances between all pairs of items. Finally, it uses translation, rotation, and reflection to obtain consistent results (Dominko and Verbič 2019). An individual item is assigned to only one cluster and colours are used to distinguish between different clusters. The size of the circle indicates citation strength or occurrence strength in the case of keywords. A more detailed account of the VOSviewer software package can be found in van Eck and Waltman (2010, 2014b). We complemented VOSviewer with CitNetExplorer, which allowed the visualisation of publications on a map where closeness between publications is highlighted on the horizontal axis and the year of publication on the vertical axis. As such, it is an ideal tool for the analysis of the development of a research field. A detailed explanation of CitNetExplorer is available in van Eck and Waltman (2014a).

To interpret the obtained bibliometric networks, items displayed on the maps were looked up in the database, allowing us to identify the topics of the items in question and the journals or proceedings in which they were published.

## Results

### Publications and citations over time

**Figure 2.**
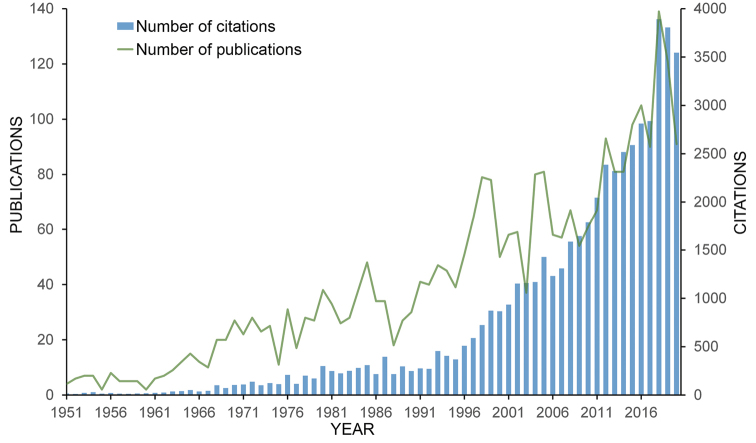
Annual numbers of articles and citations on terrestrial isopods between 1950 and 2020, obtained from Web of Science (WoS).

Our search returned 2946 items related to terrestrial isopods in Web of Science (WoS) and a total of 52631 citations (34880 excluding self-citations). At the beginning of the time period covered by our study in the 1950s, the annual numbers of publications on terrestrial isopods indexed in WoS were fewer than ten and the annual numbers of citations were below 5 (Fig. 2). The publication rate began growing in the 1960s and by 1970 exceeded 20 publications annually. Until the late 1990s, this growth was linear, with annual publication numbers increasing by ca. ten publications every decade. During the last two decades, however, the rate of publishing increased, doubling from ca. 60 items per year in the early 2000s to roughly 120 per year at end of the 2010s.

The annual numbers of citations of these works followed a similar trend, but citation numbers increased at greater rates than publication numbers. While the annual numbers of citations remained below 30 until the end of the 1960s, the frequency of citation grew steadily to ca. 200 citations per year during the 1970s. At this point, the citation rate reached a plateau and remained unchanged until the mid-1990s. After this point, the annual numbers of citations began to increase rapidly, growing by ca. 1000 annual citations every ten years (Fig. 2).

### Numbers of publications according to type

The great majority of indexed publications, more than 88%, are journal articles (Table 1). Reviews account for 3.5%. A surprisingly large percentage of publications is derived from scientific meetings; proceedings papers and meeting abstracts together account for more than 7% of all publications, and proceedings papers are the second most numerous publication type, representing almost 4% of all publications.

### Geographical distribution of research on terrestrial isopods

If we take into account publications indexed in WoS, the largest output in the study period comes from the United States with 372 publications. France is not far behind with 357 publications, followed by Germany and the United Kingdom with more than 200 publications each. Other countries with research outputs exceeding 100 publications are Italy, Japan, the Netherlands, Brazil, Slovenia and Canada (Table 2). The majority of the top ten countries in the field are G7 countries, the seven major developed economies (United Nations 2020). This is not surprising as they generally have great research outputs and investments in research and development (National Science Board 2019). The three remaining countries are Brazil, the Netherlands, and Slovenia, a more surprising group. Other prominent countries in the field (Suppl. material 1) include Tunisia, Israel, several members of the European Union, and large developing economies, such as Russia and China. When comparing the productivities of different countries, we should also consider the language bias of international scientific databases. In some countries, a large part of the research output may not be indexed, which may result in an overrepresentation of English-speaking regions (Amano et al. 2016).

**Table 1.** Numbers of publications on terrestrial isopods in WoS according to publication type.

Publication type	Number	% of all publications
Article	2597	88.1%
Proceedings paper	111	3.8%
Review	104	3.5%
Meeting abstract	102	3.5%
Note	50	1.7%
Letter	43	1.5%
Other	62	2.1%

**Table 2.** Numbers of publications on terrestrial isopods by country. The table lists the top ten countries according to the numbers of publications in WoS. These are also the countries with more than 100 publications in the dataset.

Country	Number of publications	% of all publications
United States of America	372	12.63%
France	357	12.12%
Germany	280	9.50%
United Kingdom	270	9.17%
Italy	197	6.69%
Japan	173	5.87%
Netherlands	135	4.58%
Brazil	126	4.28%
Slovenia	122	4.14%
Canada	103	3.50%

### Most productive organisations

Almost all among the ten most productive organisations in the field of terrestrial isopod biology (Table 3) are located in the ten most productive countries (Table 2). The exception is the University of Aveiro from Portugal, the eleventh most productive country (Suppl. material 1). The most productive organisation is the University of Poitiers, contributing well over 6% of all publications during the study period. Another French institution among the top 10 is the National Centre for Scientific Research, contributing approximately 1.5% of publications. Italy also has two organisations among the top ten: the National Research Council is the fourth most productive organisation, while the University of Catania took tenth place. The University of Ljubljana (Slovenia) and Vrije Universiteit Amsterdam (the Netherlands) contributed just under 4% of publications each, making them the second and third most productive organisations, respectively.

Considering the contributions of individual organisations, we can deduce that in small countries, such as the Netherlands, Slovenia, Portugal and Tunisia, almost all publications on terrestrial isopods were produced at a single institution. In Italy and France, a few dominant organisations contributed the bulk of the total research output. The same conclusion can be made for most countries outside Europe, such as Brazil, where a handful of institutions contributed the majority of publications (Suppl. material 2). This contrasts with the USA, UK, Japan and Canada, which are among the most productive countries, yet individual institutions do not stand out (Table 2 and Suppl. material 2). This suggests that research on terrestrial isopods in these countries is more dispersed among institutions and not linked to the traditions of individual institutions to the same extent as in Mediterranean countries. A similar conclusion can be reached for Germany; while Ulm University stands out in terms of productivity, it nevertheless represents only a fifth of the total German output in this field, with numerous other institutions contributing the remaining publications (Suppl. material 2).

**Table 3.** Top ten organisations that contributed the most publications in WoS in the field of terrestrial isopod biology.

Organisation	Country	Number of publications	% of all publications
University of Poitiers (Université de Poitiers)	France	196	6.6%
University of Ljubljana (Univerza v Ljubljani)	Slovenia	116	3.9%
Free University of Amsterdam (Vrije Universiteit Amsterdam)	The Netherlands	107	3.6%
National Research Council (Consiglio Nazionale delle Ricerche)	Italy	83	2.8%
University of Tunis El Manar (Université de Tunis El Manar)	Tunisia	73	2.5%
University of Aveiro (Universidade de Aveiro)	Portugal	57	1.9%
Ulm University (Universität Ulm)	Germany	53	1.8%
Federal University of Rio Grande do Sul (Universidade Federal do Rio Grande do Sul)	Brazil	53	1.8%
National Centre for Scientific Research (Centre national de la recherche scientifique)	France	40	1.4%
University of Catania (Università degli Studi di Catania)	Italy	36	1.2%

### Historic overview

In order to determine the impact of individual publications and which research topics relating to terrestrial isopods were continuously prominent, we conducted a co-citation analysis, identifying the publications most often cited by the publications in our database, regardless of whether or not the cited works were included in our WoS-derived dataset. This allowed us to identify relevant works published before 1950 and those published in non-indexed publications. As a result, the period in which these works were published is considerably longer, including most of the 20^th^ century and the first two decades of the 21^st^ century. While many more works on terrestrial isopods were published during this period (see Schmalfuss and Wolf-Schwenninger 2002), our analysis is limited to a few hundred most impactful studies for the sake of clarity.

The visualisation of the co-citation network of the 200 most frequently cited works allows us to discern six major clusters, corresponding to major topics in terrestrial isopod research (Fig. 4). One cluster, shown in purple in Fig. 4, connects works on terrestrial isopod systematics. This cluster is well connected particularly with the cluster of works on woodlouse ecology, physiology and behaviour (shown in red). This broad topic interconnects intensively with another cluster of predominantly physiological studies that focus mostly on digestive physiology and leaf litter decomposition (dark blue). It is not surprising that this digestion research is intimately linked with the green cluster, which connects works on heavy metal accumulation in woodlouse tissues and ecotoxicology. Another cluster, shown in yellow, represents studies dealing with the woodlouse exoskeleton and biomineralisation; this cluster connects with other topics to a lesser extent. Even more distant is the cluster of works dealing with microbe-host interactions, particularly on the feminising bacterium *Wolbachia* (light blue).

**Figure 3.**
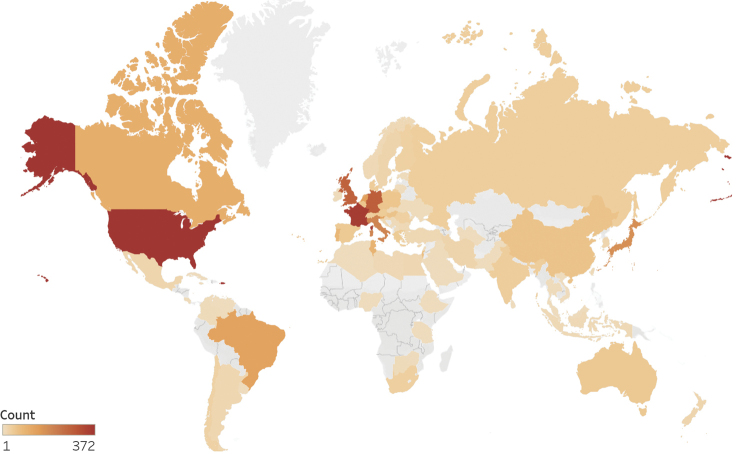
World map of terrestrial isopod research. The colour of each country corresponds to the number of publications on terrestrial isopods in WoS produced by researchers active in that country. Gray areas represent territories without publications in the study dataset.

**Figure 4.**
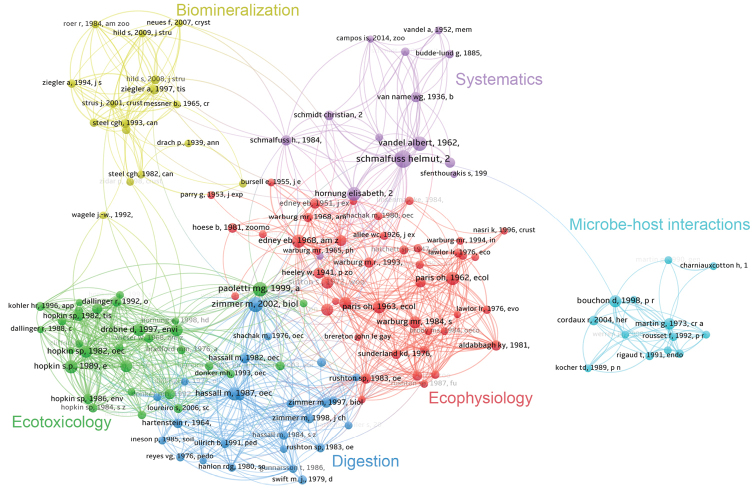
Co-citation network of 200 publications most frequently cited by works in the study dataset. Six major topics of research are discernible (shown in different colours).

**Figure 5.**
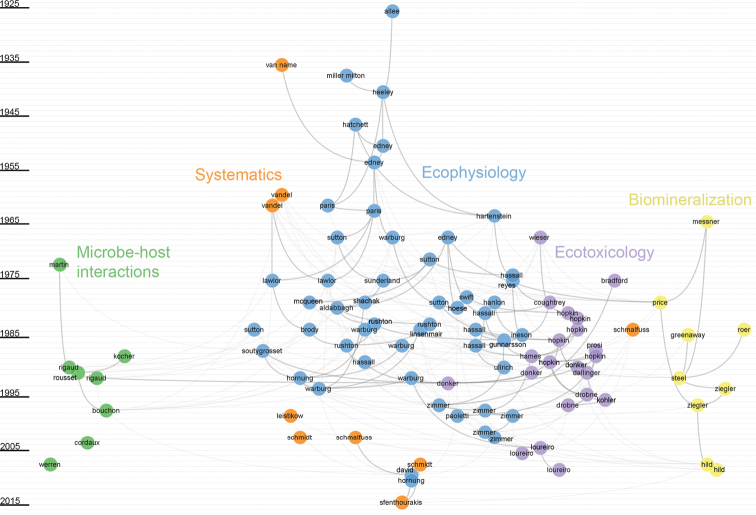
Co-citation network showing the 100 publications that were the most frequently cited by studies in the dataset. The publications are shown along a timeline spanning the period 1925–2015. Names of first authors identify publications.

By considering a subset of the 100 most influential publications along a timeline, we can identify publications that are predecessors and successors in continuous topics in the field using CitNetExplorer (Fig. 5). On the basis of co-citation, we can identify several clusters of publications that correspond to the major topics of terrestrial isopod research. Before the 1960s, the most impactful publications fall into two clusters (Fig. 5); one (shown in orange) connects works in systematics, while the other (shown in blue) represents works in ecophysiology and encompasses both physiological clusters identified in the broader analysis presented in Fig. 4. In the 1960s, terrestrial isopod research diversified and several additional clusters are identifiable in the co-citation network (Fig. 5) The cluster shown in purple is closely linked to the ecophysiological cluster and represents research on ecotoxicology; another cluster, shown in yellow, deals with biomineralisation, while the green cluster corresponds to works on microbe-host interactions, particularly on *Wolbachia*.

The systematics cluster is relatively scattered and works within it do not interconnect very frequently with each other, suggesting that they are not often cited together. Furthermore, relatively long time intervals elapsed between these works (Fig. 5). An interesting publication is the work of Schmalfuss (1984), which is a part of the systematics cluster but is very distant from other works in this area and instead occupies a central position in relation to most other clusters, establishing numerous co-citation links with them (Fig. 4). This study described the various ecomorphological types of terrestrial isopods and set a framework for numerous comparative studies in other topics. The two volumes of the monograph on terrestrial isopods prepared by Vandel (1960, 1962) hold a similar position. These works presented the morphology of numerous isopod species in great detail. In addition, they also reported on other aspects of isopod biology and thus influenced various research topics in this field.

Numerous links are obvious between systematics and ecophysiology and particularly between this cluster and ecotoxicological publications. By contrast, the clusters dealing with microbe-host interactions and biomineralisation are again more distant from other clusters, which is consistent with the results of the broader co-citation analysis (Fig. 4).

### Journals

**Figure 6.**
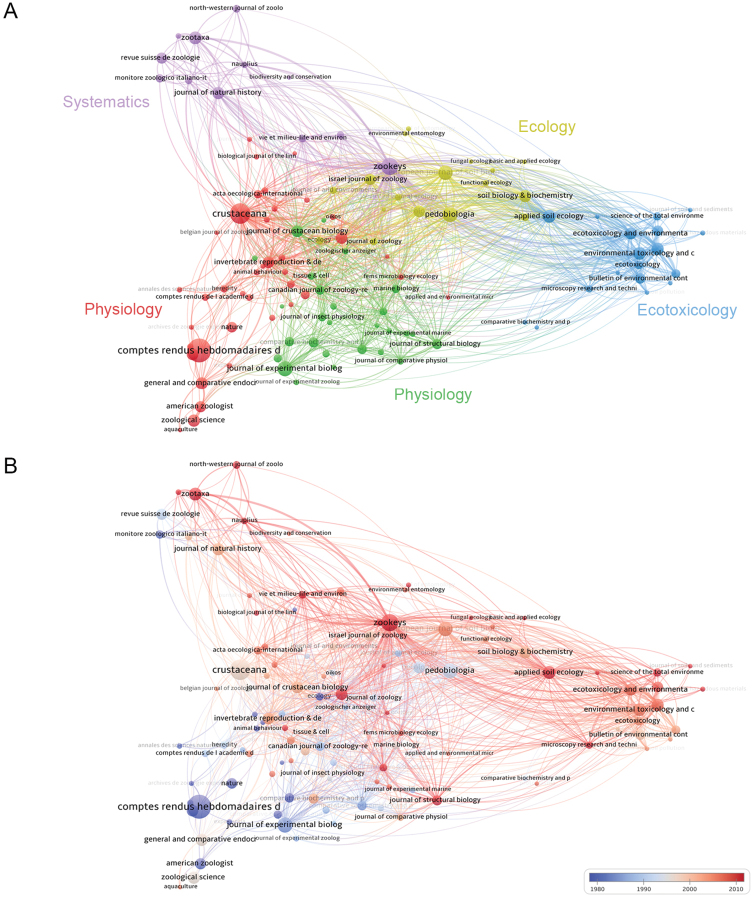
Bibliographic coupling network of journals that have been cited by publications in the database at least 50 times **A** clustering of journals, revealing several major topics that they cover **B** heat-map of the network presented in **A** showing the average year of publication of articles published in these journals and cited in the database.

The topics of journals with the most publications relevant to the field of terrestrial isopod biology fall into five major clusters on the basis of bibliographic coupling (Fig. 6A). The purple cluster encompasses journals that published largely taxonomical and phylogenetic studies in this field. The blue cluster links journals publishing articles on ecotoxicology, while the yellow cluster covers more purely ecological topics. The two remaining clusters, shown in red and green, both cover physiology; while different topics are not very sharply delineated in this case, most of the studies on biomineralisation and exoskeletal features can be found in journals in the green cluster; the red cluster, on the other hand, encompasses journals that published more works on endocrinology and microbe-host interactions. Understandably, the two physiological clusters are highly interconnected.

Considering the average year of publication of the articles, journals in the ecotoxicological cluster stand out: most cited publications in this area have been published recently (Fig. 6B). The remaining clusters display shifts from certain journals to others during the analysed period. In some of these clusters, such as the ones covering systematics and physiology, a shift is noticeable from regional to international journals. French periodicals were particularly influential in the 20^th^ century; the proceedings of the French Academy of Sciences in Paris (‘Comptes rendus hebdomadaires des séances de l’Académie des sciences’) and its later offshoot ‘Serie D’ account for 4% of all publications on terrestrial isopods, more than any other journal (Table 4). While this periodical published the largest body of isopod-related research in the 20^th^ century, its focus later shifted elsewhere.

**Table 4.** Ten journals that published the most publications on terrestrial isopods during the study period. The entry for the French periodical ‘Comptes rendus hebdomadaires des séances de l’Académie des sciences’ includes articles published in its ‘Serie D’.

Journal title	Published items	% of all items
Comptes rendus hebdomadaires des séances de l’Académie des sciences	120	4.1%
Crustaceana	89	3%
ZooKeys	66	2.2%
Journal of Experimental Biology	49	1.7%
Pedobiologia	48	1.6%
European Journal of Soil Biology	47	1.6%
Applied Soil Ecology	37	1.3%
Environmental Toxicology and Chemistry	37	1.3%
Zootaxa	35	1.2%
Zoological Science	34	1.1%

Apart from ecological and ecotoxicological journals, journals covering systematics, such as ‘Zootaxa’ and ‘ZooKeys’, have been very active in the new millennium (Fig. 6 and Table 4). The latter journal publishes thematic issues (Štrus et al. 2012; Taiti et al. 2015; Hornung et al. 2018) dedicated to terrestrial isopod biology in connection with ISTIB (International Symposium on Terrestrial Isopod Biology). This triannual meeting has been bringing terrestrial isopod researchers together regularly since 1983, with some complications only due to the COVID-19 pandemic.

These are the periodicals that published the most articles on the subject of terrestrial isopods, but which periodicals published the most influential studies? When considering the 100 most influential publications (Fig. 3 and Suppl. material 3), the article with the highest citation score was published in ‘Stuttgarter Beiträge zur Naturkunde, Serie A’, a periodical published by the Stuttgart State Museum of Natural History (Staatliche Museum für Naturkunde Stuttgart). This journal is currently published under the title ‘Integrative Systematics’. This periodical is not indexed in WoS and does not have an impact factor. Among other works with the highest citation scores, eleven have been published in the journal ‘Oecologia’. These are mostly publications found in the ecophysiological and ecotoxicological clusters identified in the co-citation analysis (Fig. 3). The next most important serial publication, ‘Symposia of the Zoological Society in London’, contributed six articles to the top 100. All six were published in a single publication, the 1984 Proceedings of the First International Symposium on Terrestrial Isopod Biology, making the first ISTIB, which took place in London in 1983, likely the most influential scientific meeting in terrestrial isopod research. These papers set the framework for a large part of studies on isopod biology over the next 40 years. They deal with a variety of topics and are assigned to several clusters in the citation network revealing major topics in woodlouse biology (Fig. 5). Like the most influential journal article, the most influential proceedings papers were thus published in a publication that is not indexed in WoS.

Among other articles in the top 100, three journals published four articles each: ‘Canadian Journal of Zoology’, ‘Environmental Pollution’, and ‘Journal of Animal Ecology’, while two journals contributed four articles each: ‘Soil Biology and Biochemistry’ and ‘American Zoologist’.

### Keywords

**Figure 7.**
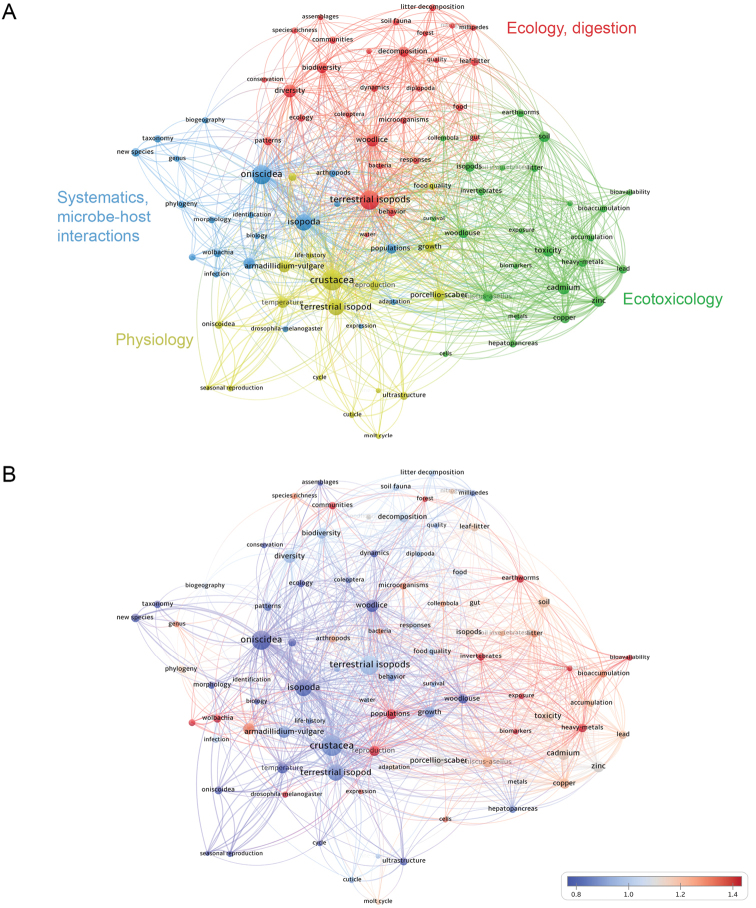
Bibliographic coupling network of keywords in publications cited in the database **A** clustering of keywords according to bibliographic coupling **B** heat-map of keywords illustrating the frequency of their citation - the citation score of an individual keyword is divided by the mean citation score of all keywords (citation scores above 1 indicate higher than average citations).

A network of 100 keywords that appeared in the database most often is presented in Fig. 7, together with a heat-map representation of how frequently articles that included them were cited (Fig. 7B). Keywords fall into five clusters, which correlate to some extent with the study areas identified by co-citation analysis (Figs 4, 5): ecology (red), ecotoxicology (green), systematics with microbe-host interactions (blue), and physiology (yellow). Links are more numerous between keywords related to ecology and ecotoxicology, while keywords relating to systematics, host-microbe interactions and physiology link to other keywords less frequently. Interestingly, keywords linked to microbe-host interactions cluster with systematics keywords, which might be due to the importance of the phylogenetic context to studies on microbe-host interactions. The association between keywords related to microbe-host interactions and the isopod *Armadillidiumvulgare* (Fig. 1A) is evident, as is the link between ecotoxicology and physiology with *Oniscusasellus* and *Porcellioscaber* (Fig. 1B, C), likely due to the long-standing tradition of these species as experimental organisms in the respective fields. As can be seen in the citation heat-map, studies on these particular woodlice were cited relatively often. The keywords occurring most frequently refer to the taxon that the work was about, e.g., “terrestrial isopods,” “woodlice,” and “Oniscidea.” These keywords occupy central positions in the network, but as can be deduced from the citation heat-map (Fig. 7B), they do not often occur in cited research, possibly due to their generality and the relatively large number of synonymous terms that label the taxon.

Keywords relating to ecotoxicology are consistently frequently cited (Fig. 7B). Among other topics of terrestrial isopod research, keywords linked to microbe-host interactions are highly cited, as well as those dealing with populations, species communities and reproduction. Apart from these, there are some highly cited keywords relating more generally to invertebrates. These differences in the citation frequencies of keywords lead us to conclude that ecotoxicology, community ecology, and microbe-host interactions are very impactful, likely due to their significance outside the realm of terrestrial isopod research.

## Discussion

Our study provides a concise overview of the development of terrestrial isopod research during the last 70 years in terms of how the extensive bibliography on this subject is structured according to publication types and the geographical distribution of publication output.

While the top countries in terms of research output include the leading developed economies with large research expenditures, which can be observed in other scientific fields as well (Holmgren and Schnitzer 2004; Jarić et al. 2012), several smaller and somewhat surprising nations are close to the top, such as Portugal, Slovenia, and Tunisia (Table 2). The high productivity of these smaller countries is linked to a handful of institutions with decades-long traditions in this area of research, as can be deduced if these results are considered alongside data on the most prolific organisations (Table 3) and the historic overview by Schmalfuss (2018). This historic survey is also an excellent source of information for all who are interested in research groups and individuals that made exceptional contributions to the field. Considerable outputs are also produced in BRIC countries: Brazil, Russia, India, and China. France has a prominent position in this field and has been home to some of the most productive researchers and organisations as well as the central journals on the subject in the 20^th^ century. Nevertheless, the majority of the French output is produced by only a few organisations with long traditions in the field, similar to the situation in smaller nations.

The language bias of international scientific databases may distort the representation publications in languages other than English (Amano et al. 2016). This becomes evident in the case of terrestrial isopod biology if we compare the outcome of our analysis of the WoS dataset with the publications published between the years 1850 and 2000 collected by Schmalfuss and Wolf-Schwenninger (2002). Our search in WoS returned fewer than ten publications in individual languages other than English and French (not shown), yet many more such publications can be found in the collection of Schmalfuss and Wolf-Schwenninger (2002) which includes non-indexed publications in a variety of languages. While this does not invalidate our identification of the group of leading nations in the field during the study period, as most of these nations are in a similar position in this respect, the numbers of publications for several countries and institutions are certainly greater than represented in WoS.

By analysing bibliometric networks, we were able to identify several major topics in terrestrial isopod research, the past and current development, and the relationships between these topics. Keyword analysis, co-citation analysis, and bibliographic coupling have identified roughly the same set of general research topics in research related to terrestrial isopods. These are: (1) ecotoxicology, (2) systematics, (3) microbe-host interactions, (4) ecology, with a great focus on population ecology and life histories, and (5) physiology. In the last field, the dominating topics are digestive physiology, ecophysiology (particularly in relation to evolutionary transition to land), and biomineralisation. Many of these topics, such as ecotoxicology, microbe-host interactions, and life-history ecology, have been identified as major topics in this field by authors who reviewed work on terrestrial isopod biology (Hassall et al. 2005; Hornung 2011; Schmalfuss 2018), whereas some prominent topics, such as ecotoxicology, were not in the scope of those reviews. This is understandable, as these areas of research do not focus on isopod biology but use woodlice as experimental organisms.

Ecotoxicology is an obvious topic on the rise. This is attested by the dominance of this field when it comes to citations and the prominent increase in the number of papers on terrestrial isopods that are published in ecotoxicological journals (Figs 6, 7). The explanation of this success is straightforward, as the field is one of the current priorities of research and development policies, particularly in Europe (European Commission 2020). As we can deduce from the obtained co-citation networks, the initial study influencing this field was the work of Wieser (1968) that deals with the metabolism of metals in the terrestrial environment, largely from the point of view of terrestrialisation in arthropods. The initial research therefore considered how isopods obtain and conserve metals as micronutrients in the terrestrial environment, in which direct uptake from seawater is impossible. This resulted in the use of woodlice as bioindicators of potentially toxic metals, leading to their extended use in ecotoxicology (Hopkin, 1989). While metal toxicity was the major focus of research at the turn of the millennium, emerging contaminants, such as nanomaterials and microplastics, are now gaining importance (summarised in van Gestel 2012 and van Gestel et al. 2018).

A similar success story is the use of terrestrial isopods in studies on biomineralisation. Here, the initial work in the co-citation network (Fig. 5) is the extensive study on the moulting process in *P.scaber* and *O.asellus* conducted by Messner (1965). Woodlice were quickly recognised as suitable experimental animals for the study of cuticle synthesis and mineralisation due to having several convenient characteristics. Woodlice preparing to moult are identifiable by the presence of sternal calcium carbonate deposits, they moult frequently throughout their lives, and they are relatively easy to maintain and handle in the laboratory. In addition, the need to conserve calcium ions necessary for the mineralisation of their exoskeletons during the process of moulting makes them particularly interesting for the study of mineral dynamics and ion transport (reviewed in Ziegler et al. 2005).

As can be deduced from the importance of the publications and keywords relating to this subject, microbe-host interactions in terrestrial isopods have been a very influential topic as well. A large part of the success of the topic likely results from work on the association of terrestrial isopods with the feminising bacterium *Wolbachia*, which is of great importance to evolutionary biology and the ecology of microbe-host interactions. Although these studies were, to a large extent, conducted on *A.vulgare*, *Wolbachia* is of wider interest as it is capable of infecting numerous arthropods and manipulating their sex. As this is also true for many insects, *Wolbachia* can potentially be used in pest management. This organism is, of course, not the only one studied in woodlice; several commensals and pathogens have been identified in these crustaceans, as well as potential symbionts aiding in the digestion of plant material and other physiological processes (reviewed in Bouchon et al. 2016). Ultimately, research on these associations is still very intensive and facilitated by recent methodological advances in the study of microbiomes (Bredon et al. 2018, 2020).

The examples of research on ecotoxicology, biomineralisation and microbe-host interactions in terrestrial isopods show how basic research on the biology of an invertebrate group later led to the establishment of the animals in question as experimental organisms in more general, even applicative, research topics. Naturally, other topics in woodlouse research had a broader impact on human knowledge as well, as we can expect from any well-conducted scientific inquiry, and these two cases were only discussed at length here as the most obvious examples in the bibliographic networks we obtained.

Despite poor funding opportunities in the field of invertebrate systematics (Martin 2011), there is vibrant evolutionary and taxonomical work on terrestrial isopods being conducted globally, which is reflected in the popularity of systematics-related journals in this field and the universal presence of a systematics cluster in the bibliometric networks obtained in this study. Furthermore, there is no apparent negative trend in the output or the impact of such studies (Figs 5, 6, 7). The relatively scarce knowledge of the diversity of woodlice, particularly in the tropics, the importance of this group from the point of view of animal terrestrialisation (Sfenthourakis and Taiti 2015; Taiti 2017), and perhaps the captivating nature of terrestrial isopods may be factors contributing to the vitality of their systematics.

As demonstrated by the co-citation analysis, the central publications in the field of terrestrial isopod biology during the last 50 years were often conference proceedings and articles published in institutional periodicals, many of which are not indexed in WoS or Scopus and lack impact factors. While the importance of scientific meetings is diminishing and conference proceedings resonate only briefly in most scientific fields (Lisée et al. 2008), symposia on terrestrial isopod biology have a large and lasting impact that is reflected in meetings contributing the most influential publications. At this point, it is too early to make any conclusions about whether or not this trend has continued in the last decade, but proceedings papers are evidently still important to the field, as can be deduced from the intensive bibliographic coupling of the journal that publishes them (Fig. 6). The ultimate importance of proceedings papers and articles in local periodicals shows that as far as terrestrial isopod biology goes, journal metrics do not likely reflect the influence that the publications ultimately have. By extension, the impact factors of journals in which invertebrate zoologists publish their work are poor predictors of the impact these researchers have on their field, a fact we fear is insufficiently appreciated.

## Conclusions

The findings of this study point out that publishing in invertebrate zoology follows somewhat specific principles, with great importance of in-person meetings and publications not captured by conventional bibliographic metrics. In addition, major contributions in this field are produced in small and developing economies at institutions that can achieve continuity of research on a topic despite changing research trends. Ultimately, the example of terrestrial isopods shows that basic research on the biology of a seemingly marginal group of invertebrates can lead to flourishing applicative research on some of today’s most pressing issues. This is all the more reason not to neglect such research in the future, as the findings of today can help resolve the issues of tomorrow.

## Supplementary materials

Supplementary material 1 Number publications according to country Data type Bibliometric data. Explanation note A table listing countries with corresponding numbers of publications on terrestrial isopods in the period between 1950 and 2020. https://binary.pensoft.net/file/684886This dataset is made available under the Open Database License (http://opendatacommons.org/licenses/odbl/1.0/). The Open Database License (ODbL) is a license agreement intended to allow users to freely share, modify, and use this Dataset while maintaining this same freedom for others, provided that the original source and author(s) are credited.Miloš Vittori, Miha Dominko

Supplementary material 2 Number of publications according to institution Data type Bibliometric data. Explanation note A table listing institutions with corresponding numbers of publications on terrestrial isopods in the period between 1950 and 2020. https://binary.pensoft.net/file/684887This dataset is made available under the Open Database License (http://opendatacommons.org/licenses/odbl/1.0/). The Open Database License (ODbL) is a license agreement intended to allow users to freely share, modify, and use this Dataset while maintaining this same freedom for others, provided that the original source and author(s) are credited.Miloš Vittori, Miha Dominko

Supplementary material 3 Most influential publications on terrestrial isopods Data type Bibliometric data. Explanation note A table listing 100 publications on the subject of terrestrial isopods with the highest citation scores. https://binary.pensoft.net/file/684888This dataset is made available under the Open Database License (http://opendatacommons.org/licenses/odbl/1.0/). The Open Database License (ODbL) is a license agreement intended to allow users to freely share, modify, and use this Dataset while maintaining this same freedom for others, provided that the original source and author(s) are credited.Miloš Vittori, Miha Dominko
